# Reduction of Adolescent Idiopathic Scoliosis and Improved *Z*-Axis Alignment of the Entire Spine When Treating a Symptomatic Patient Using a Multidisciplinary Approach: A Case Report

**DOI:** 10.3389/fresc.2022.917519

**Published:** 2022-06-20

**Authors:** Juan Jesus Villa, Zhiyang Zhao, Weicheng Pan, Yongfei Guo

**Affiliations:** ^1^Xuyang Doctor Group Co., Ltd., Shanghai, China; ^2^ASPINE Health Group, Inc., Union City, CA, United States; ^3^Department of Orthopedics, Second Affiliated Hospital of Navy Medical University, Shanghai, China

**Keywords:** idiopathic juvenile scoliosis, cervical kyphosis, low back pain, ASPINE Systems, spinal XYZ traction

## Abstract

**Background:**

This study presents findings on improvements of both the X-axis and Z-axis posture in a young female with adolescent idiopathic, scoliosis suffering from pain complaints who was treated with a multidisciplinary approach.

**Case Presentation:**

The 15-year-old patient reported low back pain for several months. Full spine radiographic assessment revealed a cervical kyphosis, forward head translation, a right ribcage translation, a left higher shoulder, and a dextroconvex lumbar scoliosis with a Cobb angle of 23°. The patient was treated with novel ASPINE Systems treatment protocols incorporating posture exercises, muscle balancing exercises, spinal 3D traction, and spinal manipulation.

**Results:**

Assessment after 50 treatment sessions over 32 weeks revealed a dramatic improvement in postural distortions. The cervical kyphosis was reduced by 9° and was accompanied by a reduction in forward head posture, centering of the thoracic spine, leveling off her shoulders, and a reduction in the dextroconvex scoliosis by 10°. The lower back pain was relieved.

**Conclusion:**

A reduction of postural distortions including idiopathic adolescent scoliosis resulted from a multidisciplinary approach utilizing ASPINE Systems.

## Background

Scoliosis is defined as a shift from the normal vertical axis of the spine, consisting of a lateral curvature including rotation of the vertebrae. For scoliosis to be considered, there should be at least 10° of spinal angulation on the radiograph associated with vertebral rotation (1). Idiopathic scoliosis is a diagnosis of elimination; hence, this condition is diagnosed only when the historical, clinical, and radiological findings do not provide a clear indication for any specific cause of origin (2). Idiopathic scoliosis is extremely atypical in infancy and early childhood but has an occurrence of 1–2% among school children up to age 15 (3). It has been suggested that idiopathic scoliosis is postural; meaning that postural distortions over time affect the structure and integrity of the spine (4).

Spinal coronal curvatures, even by small degrees such as 10, at post-puberty may not rule out later development of scoliosis; due to the possible pathogenesis of later development results from “the inevitable recurring bio-mechanical tensions of daily living being applied continuously and asymmetrically to the spinal deformity,” any conservative approach to successfully better improve the scoliotic spine is beneficial regardless of age (5).

Intensive scoliosis inpatient rehabilitation (SIR) programs have been used for decades for the reduction and prevention of deformity progression (6–8). This treatment is based on a rigorous multimodality “Schroth method” program consisting of exercises, bracing, manual therapy such as spinal manipulation and acupuncture, including their signature procedure, breathing exercises (9). According to (SIR) scoliosis treatments are performed constantly in order to have favorable outcomes.

**Figure 1:**
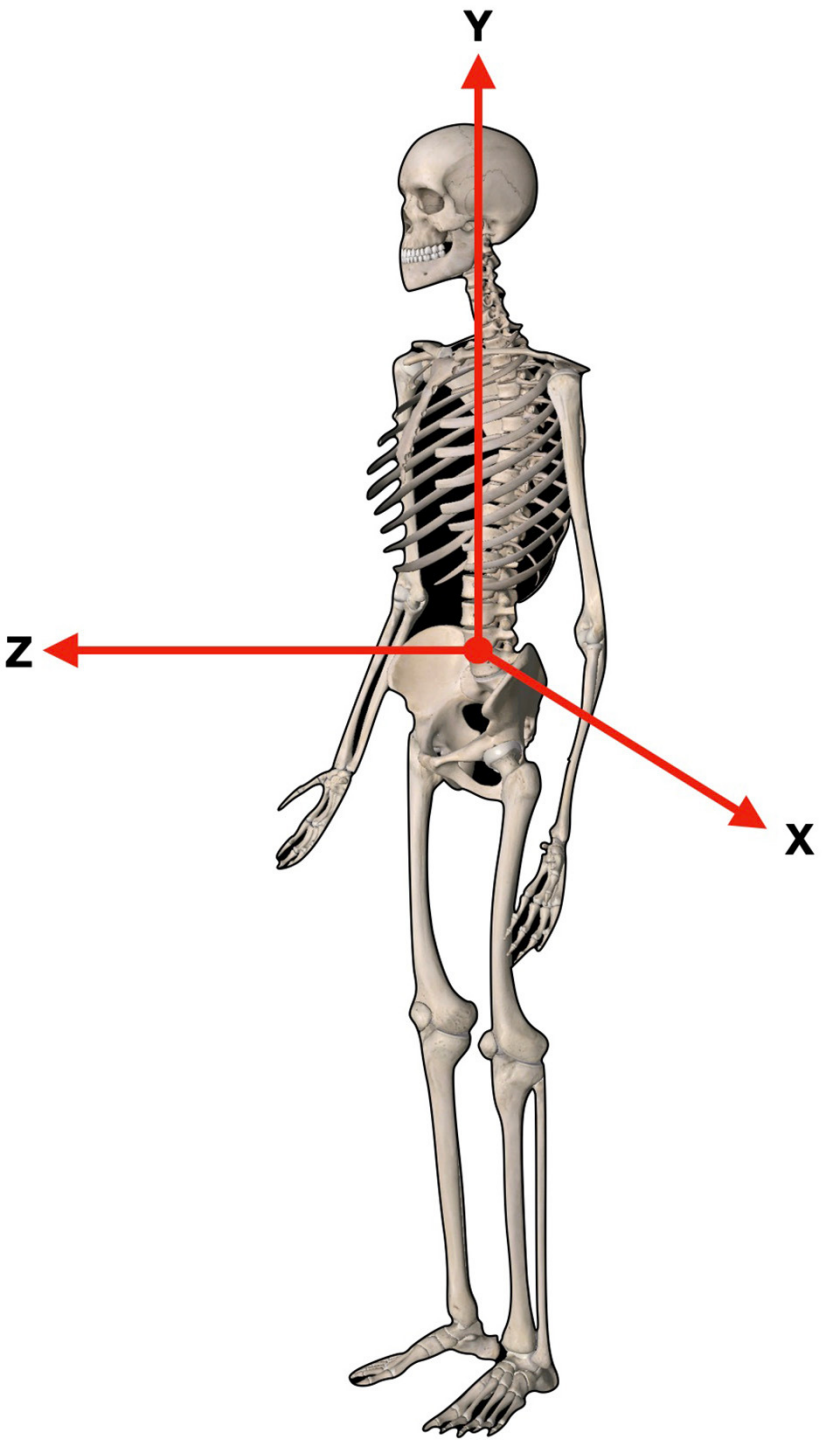
*Y*-axis represents the frontal axis, superior/inferior. *Z*-axis represents the sagittal axis, anterior/posterior. *X*-axis represents the transverse axis right to left.

The International Society of Biomechanics (ISB) suggests that human kinematics is quantified by means of an XYZ Cardan sequence of rotations and translations. X-axis represents the transverse axis, the *Y*-axis represents the frontal axis, and the *Z*-axis represents the sagittal axis, respectively (10) (Figure 1).

This case demonstrates improvements in both the *X*-axis and *Z*-axis posture in a young female with adolescent idiopathic scoliosis suffering from low back pain as treated with a multidisciplinary approach utilizing novel ASPINE System treatment protocols performed twice a week.

## Case Presentation

On August 28, 2020, a 15-year-old female presented to the clinic with the chief complaint of constant low back pain for 2 months, occurring daily and relieved with rest; but aggravated with prolonged activities like being standing or seated for more than 1 h. She initially felt low back pain 2 months prior to her coming to the clinic. She was treated prior by another spinal practitioner for two consecutive sessions using spinal manipulation, which relieved her pain for 2 weeks. However, her current low back pain episode started 4 days earlier. The patient also reported suffering from “bad posture,” which she described as, “a hunched back and rigid.” The patient rated her LBP at 5/10 on average (0 = no pain; 10 = worst pain ever) and an 8/10 at worst. The patient scored 25% on the Revised Oswestry Chronic Low Back Pain Disability Questionnaire (ODI).

During the initial physical examination the patient reported pain and tenderness on palpation of the mid-cervical spine and lumbar region, paraspinal musculature felt taut and tender. Lumbar range of motion (ROM) was limited in forward flexion of about 60% (visually estimated) due to her hypertonic hamstrings. Cervical ROM was limited in both the right and left lateral flexion by 30% (visually estimated). All other orthopedic tests were unremarkable. Several spinal segmental fixations were identified in the cervical and lumbar region *via* segmental spinal motion palpation.

The patient brought with her a full spine standing radiographic assessment that included anterior-posterior (AP) and lateral cervical, thoracic, and lumbar images. The images were assessed and digitized using novel ASPINE Systems (Shanghai, China). The ASPINE System refers to a standard protocol for spinal diagnostic procedures and non-invasive multidisciplinary rehabilitation treatments. ASPINE Systems developed a novel digitizing image data collection and X-ray interpretation software. The lateral images were calculated *via* the Harrison Posterior Tangent (HPT) method, which measures the rotation between adjacent vertebrae using lines contiguous with the posterior margins of each vertebral body (11). The AP views are assessed *via* the Risser-Ferguson method, best fit lines from the assessed center of mass of each vertebra, and the Cobb Method, the gold standard for scoliosis measurement (12, 13).

Several positive postural abnormal factors were identified including forward head shift as measured as the horizontal distance from the posterior superior C2 body to a line drawn from the posterior inferior C7 vertebral body; showed a large forward head (14). The lateral lumbar view showed an L1–L5 hyper lordosis of [−48.5° vs. −40 normal (11)]. A negative numeric value indicates the direction of the curvature; a negative value refers to a lordosis, a positive value indicates a kyphosis. Cervical lordosis from C2-C7 showed a reversal curve [+1° vs. −42° normal (11, 15)]. The AP images showed a Risser Ferguson (RF) of 22° and a Cobb of 23° in her lumbar spine.

This case study occurred during the outbreak of COVID-19. By August 2020 China had already implemented quarantine and preventative measures all over the country, meaning COVID-19 did not delay or alter the course of the treatments.

## Methods

After the initial assessment, the patient consented to treatment targeted at the correction of her altered *Z*-axis and *X*-axis posture. The patient started treatment incorporating the ASPINE Systems utilizing the data collected and analyzed to create an individualized care plan with the purpose of correcting the abnormal postural distortions. The multidisciplinary treatment approach to this case consisted of exercises to create a balanced musculature in the spine, postural spinal traction, posture corrective exercises, and spinal manipulations (11, 16).

**Figure 2:**
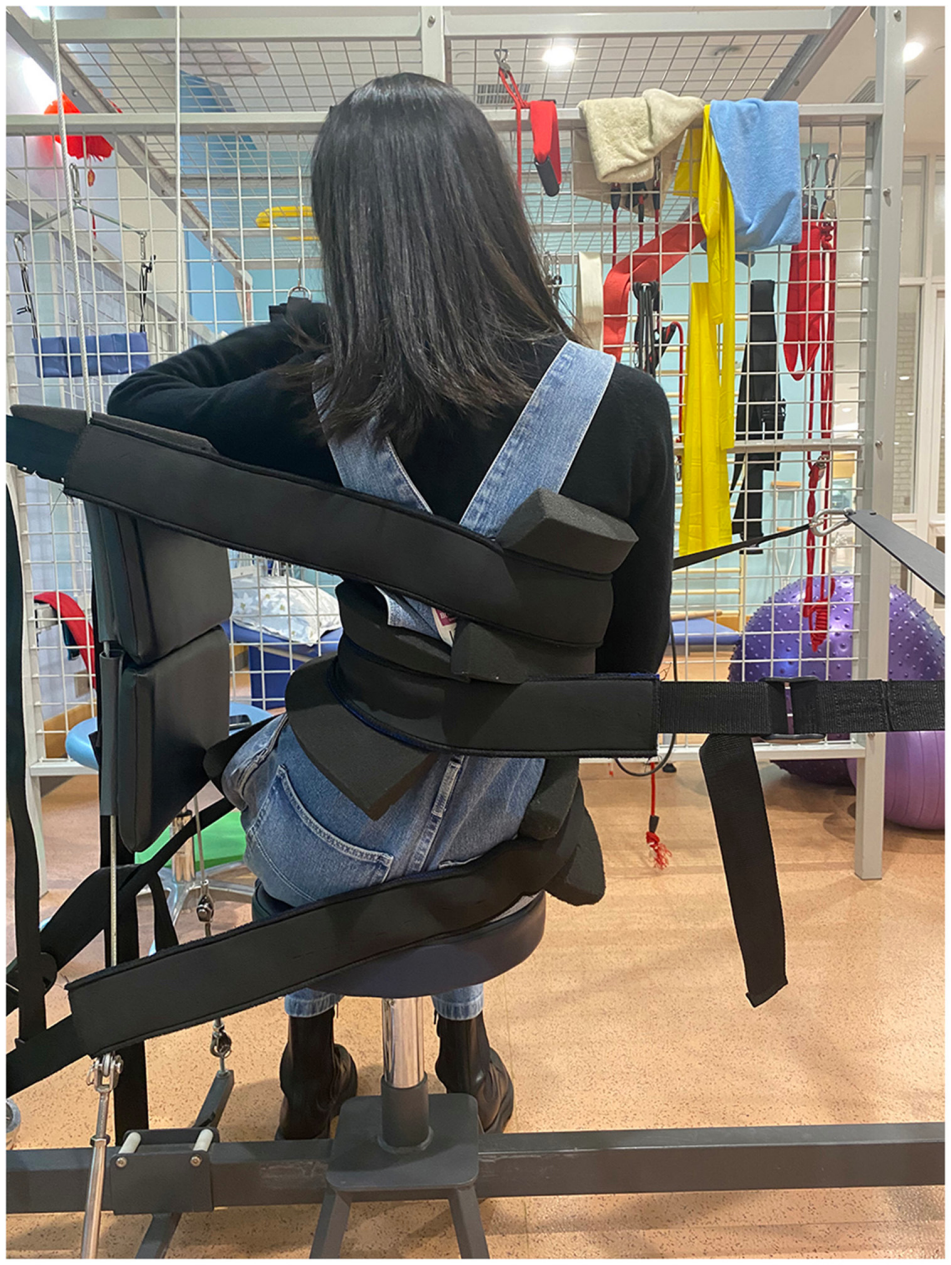
Traction set-up. Patient seated. Pelvic stabilization to prevent any pelvic displacement. Patient performed the appropriate corrective movements then L3 was pulled from the left. Her ribcage was stabilized to prevent any further displacements. Traction was performed for 20 min.

The patient performed lumbar *X*-axis traction (Figure 2) in the Spinal Traction Platform (STP) in the seated position. The patient's pelvis was stabilized preventing her pelvis from shifting. She rotated her lumbar spine to the right (-Ry) and translated her lumbar spine to the right (-Tx), creating left lumbar lateral flexion (-Rz), and a strap was placed on the left side (convex) of lumbar scoliosis.

The strap was pulled until her lumbar spine shifted to the right. A thoracic strap was used on the right side of her ribcage to stabilize her thorax. Tension was maximized to patient tolerance after every 3 min until reaching 20 min.

The patient also performed three different exercises to create spinal musculature balance, correcting the lumbar *X*-axis and the cervical *Z*-axis postural distortions. The first exercise is called the “Birddog,” where the patient is on all fours (knees bend) and lifts the right leg and left arm. This exercise aims to maintain proper core control and stabilization of the spine. A scoliosis meter (spine inclinometer) device was used to assist in further evaluation of the weak side (17). In this case, the left side (convex side of scoliosis) was weak. She started with 15 repetitions with no rest period. The process of extending the opposite side extremities to be parallel with the floor and returning to the start position was considered to be one repetition. After the fifteenth treatment, her repetitions increased to thirty.

The second exercise was called the “Lumbar Posture Exercise,” and involved the patient standing erect with feet shoulder-width apart and performing 3 distinct lumbar movements; right lumbar rotation, right lumbar translation, and left lumbar lateral flexion (-Ry, -Tx, -Rz) in this unique order while keeping their arms crossed (11, 14). Each sequence of lumbar movement was followed from the previous lumbar position. For example, after performing the right lumbar rotation, she kept the position then performed right lumbar translation, kept the position, and performed left lumbar lateral flexion. This sequence was considered to be one repetition. She performed a total of 100 Lumbar Posture Exercise repetitions with no rest period for the duration of the 50 treatments.

The third exercise is called “Neck Extensions,” which consists in bringing the head from a neutral position to an extended position using a single resistance band. One repetition was considered after the head was moved behind the shoulders and brought back to neutral. The goal was to bring her ear behind her shoulder. She did a total of fifteen repetitions initially and this was increased to thirty repetitions after the fifteenth treatment with no rest period.

The patient underwent spinal manipulative and postural therapy each session. The patient was compliant with the treatment plan that included two times a week from August 28, 2020, to August 30, 2021, for a total of 50 sessions over 32 weeks. The clinician kept a daily record of visits to ensure the patient adhered to treatment and exercises. Videos were recorded by their parents and shown to the practitioner to ensure the correct form and procedures of her exercises. There was parental consent to the publication of these results including any pictures and radiographs.

## Results

**Figure 3:**
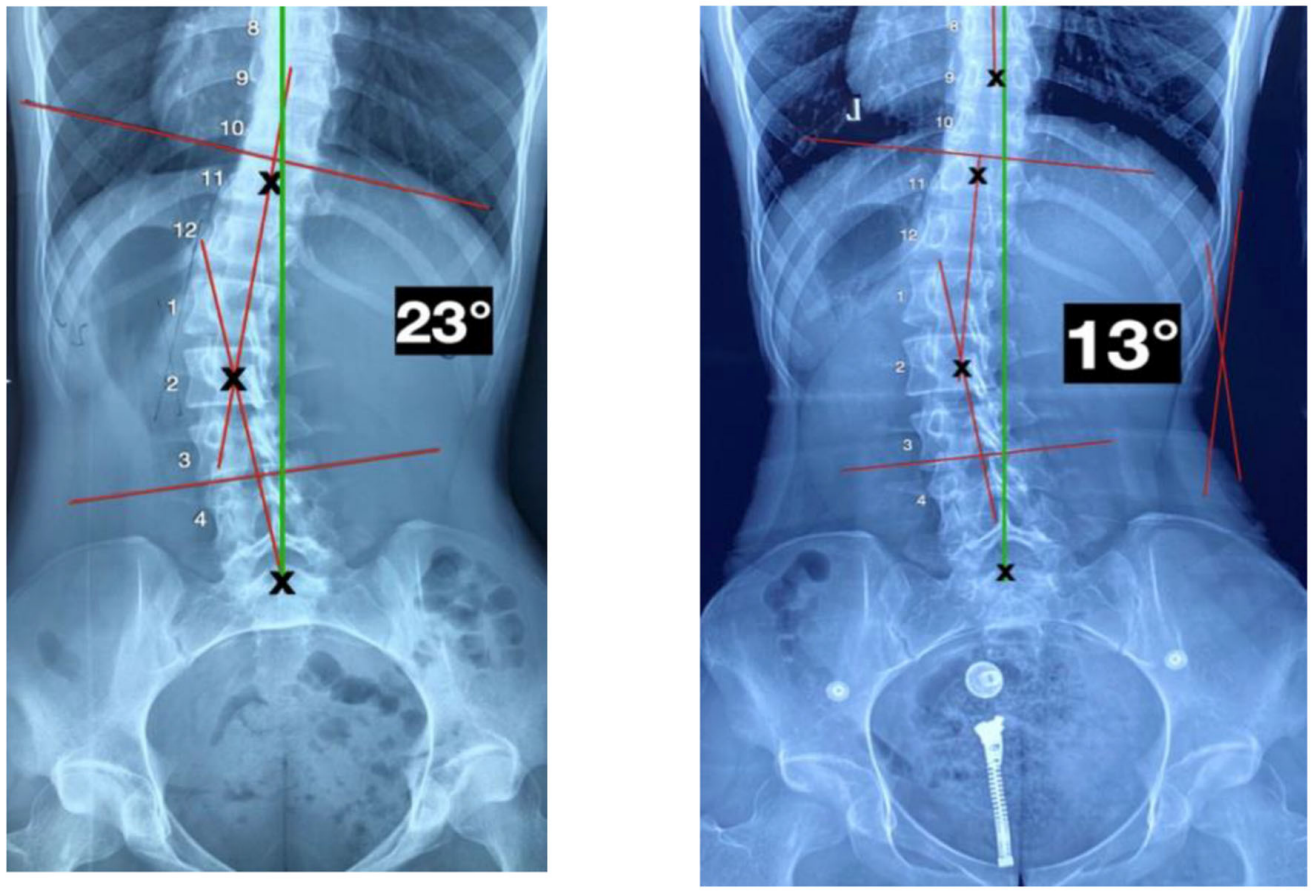
P-A X-ray views. **Left**: Initial image showing a cobb angle of 23°. **Right**: Image showing a cobb angle of 13°.

**Figure 4:**
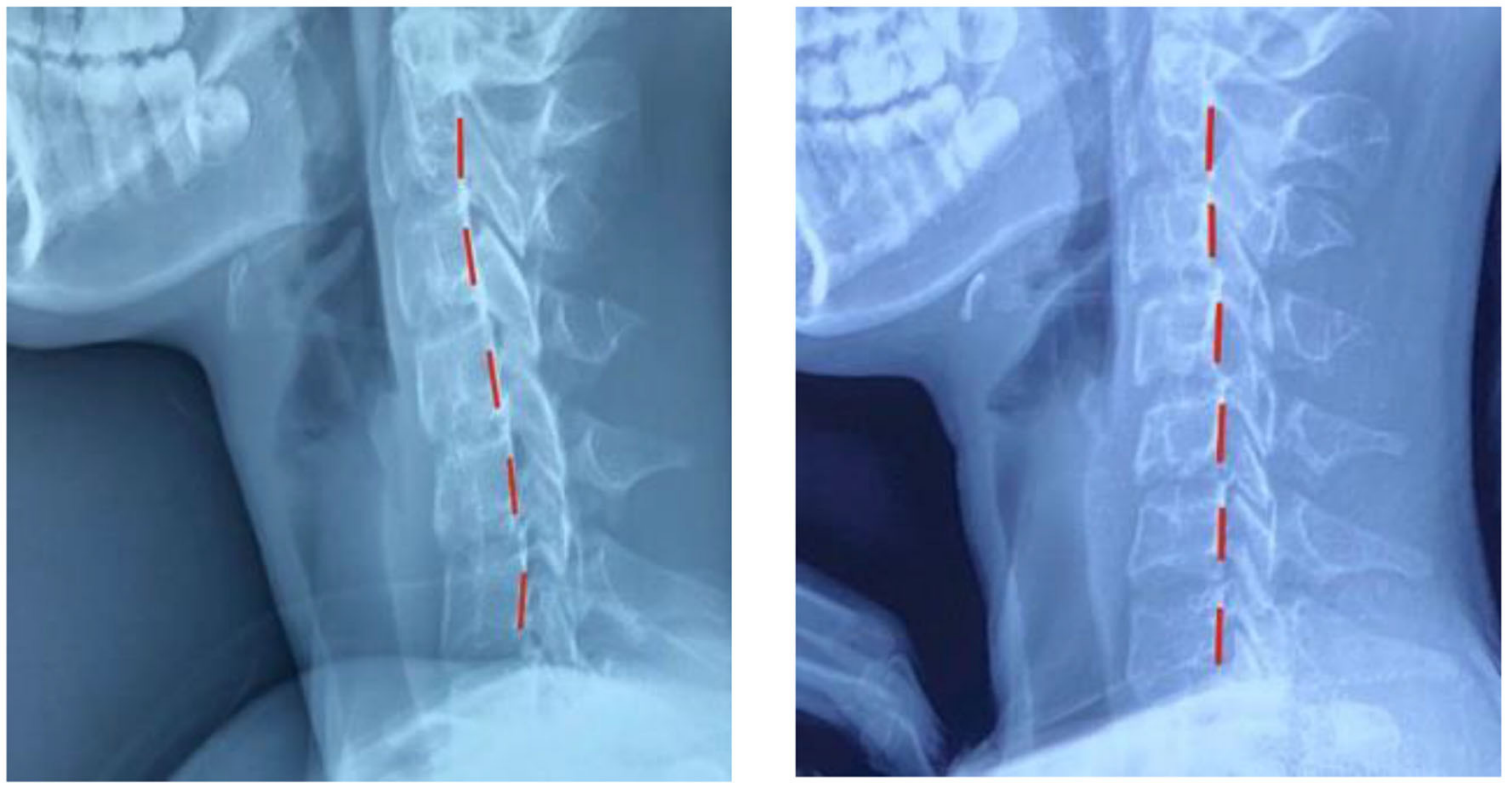
Lateral cervical views. **Left**: Initial image showing prominent forward head shift. **Right**: Reduction of forward head shift. Red line is the patient's position.

Upon reassessment after 50 treatments, the patient reported having total relief from LBP with no subsequent episodes of pain. All cervical and lumbar ROM were normal with no pain or discomfort. The patient rated her LBP at 0/10 (0 = no pain; 10 = worst pain ever). The ODI score was 0% for LBP. The patient quoted, “I do not feel pain anymore, my posture is much better, and my spine feels more flexible.” Repeated radiographic assessments showed a significant improvement in several postural parameters (Figure 3): *Z*-axis cervical curve increased to −7° (vs. +1°) a 19% difference, and the Z-axis thoracic curve decreased to +31° (vs. +40°) an 8% difference. The *Z*-axis lumbar curve increased to −44° (vs. −36°) a 20% difference. Her X-axis posture was improved in her lumbar spine with a lumbar Cobb of 13° (vs. 23°) (Figure 4).

## Discussion

This patient experienced an improvement in overall spine posture, along with a reduction in lumbar scoliosis. This is essential as postural and spinal compensations will occur as spinal deformities evolve over time. It is important to perform full-spine radiographic assessment in patients presenting with spinal deformity (including scoliosis) to properly diagnose biomechanical disorders (18), as with ongoing methods such as ASPINE Systems, the treatment is dictated by both postural radiographic measurements (9).

It has been implied that if scoliotic curves progress from asymmetric loading, the shearing force component would be the cause. This can be from a pelvic morphology or un-leveling of the spine. Therefore, any conservative correction is advantageous, as the spine without scoliosis will be subject to fewer shear forces and therefore less prone to scoliosis progression; such as heel lift or pelvic Y-axis manipulation (19).

SIR programs are rigorous with constant treatments to have favorable outcomes (6–8), putting a burden on the patient's daily routines. In China, children have extreme pressure from their parents to do well in school and become the best. Their schedules are full with regular classes starting at 7:00 a.m. and finishing after 4:00 p.m. and then they attend extracurricular activities and classes to stay ahead of the rest of their classmates. Most children finish their day around 8 or 9:00 p.m.; however, they still need to do their homework. In China (SIR) is not efficient if the patient cannot adhere to the rehabilitation program. A multimodality rehabilitation program that involves less in-clinic treatments and more in-home exercises and procedures is more favorable for successful outcomes.

Standard ASPINE treatment protocols utilize data collected by posture and X-ray software to create a care plan involving spinal manipulation, corrective posture and muscle balance exercises, and multidimensional spinal traction procedures.

It is not known which contributed to the correction of the spinal postural distortion. Regardless, it was the application of the patient-specific postural correction methods that led to the following treatment and follow-up. Scoliosis treatment using a multidisciplinary approach such as combining manipulative and rehabilitative therapy has shown efficacious results in similar cases (20). Since spinal manipulation has proven ineffective in reducing scoliosis curvature (14), further study in these methods is recommended to evaluate different postural combinations and results obtained in a larger population of scoliosis patients using a multidisciplinary approach.

Limitations to this case are that this is only a single case. A further limitation is that there is no long-term follow-up. Other limitations include the lack of constant radiographic reproduction. In China, each hospital sets its own rules and regulations for its procedures. There is no governmental agency that oversees medical procedures. For this reason, consistent radiographic imaging reproduction is extremely difficult to come by. Additional limitations to this case still involve radiography. It is common in China to use plain films when analyzing radiographical imaging. The ordinary film poses challenges, as the quality of the film can sometimes prevent suitable post-processing parameter measurements.

## Conclusion

A reduction of postural disorders including idiopathic adolescent scoliosis resulted from a multidisciplinary approach, incorporating spinal manipulation, muscle balancing rehabilitation, and postural traction; performed twice a week. In this case, the patient had a 10° reduction in her scoliosis in 32 weeks after 50 treatments. Further studies incorporating a multimodality rehabilitative regimen in scoliosis cases are suggested. This case study incorporates novel ASPINE Systems and protocols. The same protocols of spinal correction using spinal manipulation, corrective exercises, and spinal multidimensional traction can be applied for further research to validate ASPINE Systems as an accepted spinal rehabilitation program.

## Data Availability Statement

The original contributions presented in the study are included in the article/Supplementary Material, further inquiries can be directed to the corresponding author/s.

## Ethics Statement

The studies involving human participants were reviewed and approved by Yunlin Rehabilitation Hospital. Written informed consent to participate in this study was provided by the participants' legal guardian/next of kin. Written informed consent was obtained from the individual(s), and minor(s)' legal guardian/next of kin, for the publication of any potentially identifiable images or data included in this article.

## Author Contributions

JV and ZZ designed the research study. WP analyzed the data. JV wrote the paper. YG gave a substantial contribution to the acquisition of the data. All authors approved the final version.

## Funding

This study was funded by the Department of Orthopedics, Second Affiliated Hospital of Navy Medical University, Shanghai.

## Conflict of Interest

JV was employed by Xuyang Doctor Group Co., Ltd. The remaining authors declare that the research was conducted in the absence of any commercial or financial relationships that could be construed as a potential conflict of interest.

## Publisher's Note

All claims expressed in this article are solely those of the authors and do not necessarily represent those of their affiliated organizations, or those of the publisher, the editors and the reviewers. Any product that may be evaluated in this article, or claim that may be made by its manufacturer, is not guaranteed or endorsed by the publisher.

## Supplementary Material

Supplementary Figure 1 ASPINE Systems treatment standard protocols. Patient's data including complaints is obtained first, then a physical examination is performed for proper diagnosis. Posture and radiographic images are digitized and analyzed. The data collected is used to determine a multidisciplinary corrective care plan focusing in spinal manipulation, corrective exercises, and spinal multidimensional traction. The goal is predicable patient results.
