# Supplementary figures and images for: Reduction of Adolescent Idiopathic Scoliosis and Improved Z-Axis Alignment of the Entire Spine When Treating a Symptomatic Patient Using a Multidisciplinary Approach: A Case Report
Source: Front Rehabil Sci. 2022 Jun 20;3:917519. doi: 10.3389/fresc.2022.917519 (PMC9397792; doi:10.3389/fresc.2022.917519)

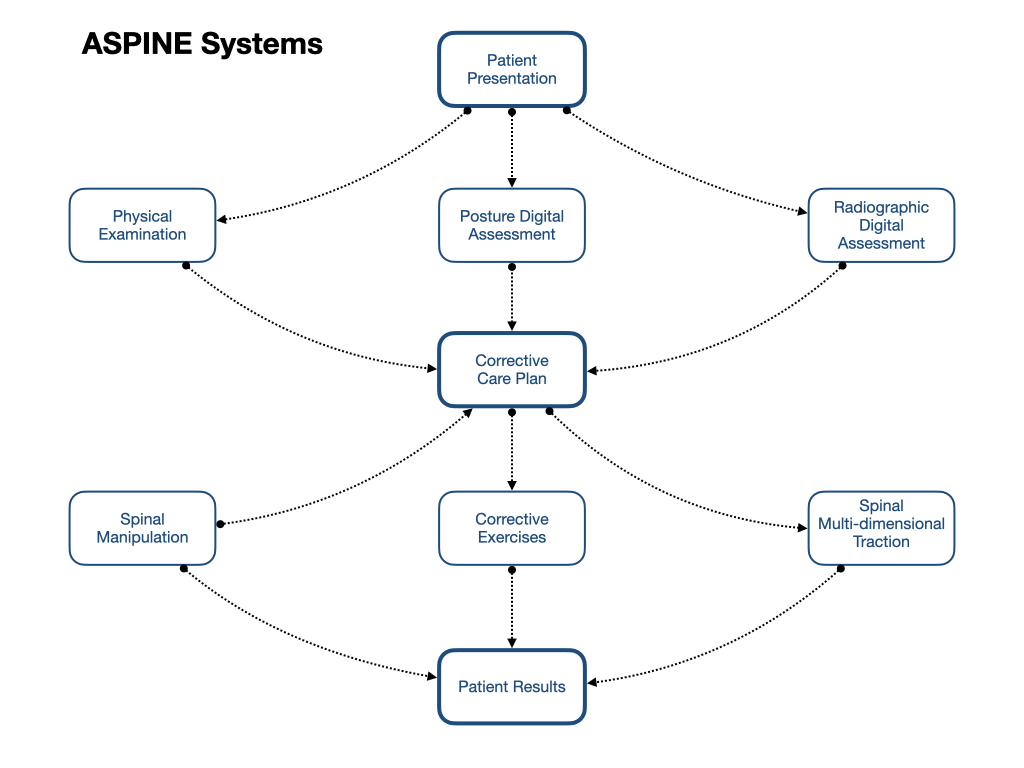

Supplement: Supplementary Figure 1 — ASPINE Systems treatment standard protocols. Patient's data including complaints is obtained first, then a physical examination is performed for proper diagnosis. Posture and radiographic images are digitized and analyzed. The data collected is used to determine a multidisciplinary corrective care plan focusing in spinal manipulation, corrective exercises, and spinal multidimensional traction. The goal is predicable patient results. [file Image_1.JPEG]
